# The Effects of the Structure and Composition of the Hydrophobic Parts of Phosphatidylcholine-Containing Systems on Phosphatidylcholine Oxidation by Ozone

**DOI:** 10.1007/s00232-017-9976-8

**Published:** 2017-08-10

**Authors:** Elżbieta Rudolphi-Skórska, Maria Filek, Maria Zembala

**Affiliations:** Department of Biochemistry, Biophysics and Biotechnology, Institute of Biology, Pedagogical University, Podchorążych 2, 30-084 Cracow, Poland

**Keywords:** Phosphatidylcholine oxidation, Ozone, Unsaturated fatty acid chains, Model membranes

## Abstract

The degree of lipid unsaturation is a parameter used to describe membrane susceptibility to oxidation. This paper highlights the importance of double bond distribution in the hydrophobic parts of lipid layers. The problem was studied by determining the effects induced by ozone dissolved in an aqueous phase acting on layers of unsaturated cholines of various molecular structures, including bi-unsaturated (DOPC), mono-unsaturated (POPC) and natural origin (soy PC). The destructive effects of ozone were quantified as the ratio of areas per molecule, which corresponded to a 1 mN/m rise in the layer surface pressure for oxidized to non-oxidized lipids (*A*_lift_/*A*_lift_^0^). The experimental results showed different behaviours among the studied lipids. Layers of DOPC with both unsaturated fatty acyl chains exhibited the greatest disruption compared with that of PC extracted from soy, which maintained stability despite high degree of unsaturation. Mono-unsaturated ozonized layers of POPC did not exhibit any disruption, but their modified properties indicated structural changes caused by the appearance of oxidation products. The stability of mixed layers (of the same unsaturation degree as the soy PC) composed of DOPC and fully saturated lipid increased, however, not reaching the soy PC level. Comparisons of the behaviour of tested systems indicated that the fraction of lipids containing one saturated acyl chain is the parameter most important for stability of the oxidized layer. The stabilizing effects of the cholesterol admixture were also quantified. Results obtained for lipid layers were supported by measurements of liposome size, zeta potential and surface tension of liposome suspension.

## Introduction

Lipid oxidation in the presence of oxidative stress may lead to severe diseases, such as cancer, atherosclerosis and Parkinson’s disease (Roberts et al. 2009; Reis and Spickett 2012). This reaction is also very important for the function of pulmonary surfactant, whose ability to decrease the surface tension within alveoli is dependent on the lipid composition. The main components of the surfactant are phospholipids (approx. 90%) with varying saturation of fatty acid residues; the major fraction comprises phosphatidylcholines (Putman et al. 1997). Ozone-polluted air coming in direct contact with the surfaces of alveoli may lead to lung tissue damage, owing to the oxidation of lipids in the surfactant and in cell membranes (Thompson et al. 2010). Toxic effects of ozone should also be considered along with the ability of oxidized lipids, including oxidized phospholipids and cholesterol (the most abundant neutral lipid present in pulmonary surfactant), to function as signal molecules.

Ozone attacks the double bonds of unsaturated fatty acids and results in the formation of ozonides, which subsequently decompose into other products, such as aldehydes and hydroxyl-hydro-peroxides. Generally, larger lipid molecules are decomposed into cleavage products with smaller molecular weights and modified substrate molecules that have completely different properties (Pryor et al. 1991). Intermediate products of fatty acid oxidation (hydroxy- or hydroperoxy-derivatives), which have sizes comparable to those of unoxidized substrate molecules and are spread on a polar subphase, form more expanded layers, owing to the hydrophilic groups situated inside the hydrocarbon chain. In addition, desorption of oxidized forms from interfacial layers has been detected in such systems (Abousalham et al. 2000). Mechanisms of fatty acid oxidation are important for the quality and conservation of food and also are crucial for the behaviour of lipids under oxidative conditions (Reis and Spickett 2012; Pryor et al. 1995).

The interfacial behaviour of oxidized phospholipids differs depending on the distribution of double bonds in the lipid hydrocarbon chains of reactants. When phospholipids have double bonds in both hydrocarbon chains, oxidation leads to the formation of smaller-size oxidation products cleaved from larger lipid molecules and of lipid residues with truncated hydrocarbon residues. The higher solubility (even volatility) of small-molecular products, allows them to dissolve in polar media (or to evaporate), thus resulting in the substantial loss of a material’s ability to form lipid structures, such as monolayers and bilayers, membranes, liposomes and vesicles. In addition, smaller-size oxidation products cleaved from substrate lipid molecules while remaining in the membrane vicinity can form other structures, e.g. reverse micelles (van Duijn et al. 1984). Oxidation products can cause effects, such as solubilisation (Khabiri et al. 2012) and adsorption and penetration, which may affect the properties of lipid structures (Qiao et al. 2013).

Additional polar groups at the locations of double bonds in the substrate molecule (inside its hydrophobic part) change the properties (e.g. polarity, charge) of larger oxidation products, thereby affecting molecular interactions. This can meaningfully modify the structures of systems composed of fully oxidized molecules or mixed with unmodified lipid molecules.

These problems can be studied either by determining the properties of mixed systems containing reaction products (i.e. mimicking the real arrangements) or by following the direct response of lipid structures exposed to oxidants.

Layer destabilization has been revealed experimentally as a significantly decreased ratio of the formal area per molecule in a layer oxidized by ozone dissolved in an aqueous subphase to the real area per molecule in a layer of the same unmodified lipid, corresponding to a 1 mN/m increase in layer surface pressure (called the lift-off areas) (Rudolphi-Skórska et al. 2014, 2016). Numerous studies have reported a loss in continuity of the strongly oxidized membranes that leads to pore creation and increased water permeability (Vernier et al. 2009; Cwiklik and Jungwirth 2010; Lis et al. 2011).

As we have previously shown (Rudolphi-Skórska et al. 2014) and also confirmed in this study, saturated phospholipids do not undergo oxidation. When phospholipid reactants are mono-unsaturated, i.e. they have one saturated hydrocarbon chain, layer stability can be maintained even after shortening the second, initially unsaturated, chain. Fragments of hydrocarbon chains cleaved at the initial locations of double bonds have been detected as a gaseous product of oxidation (Wadia et al. 2000). The groups (aldehyde, carboxylic) formed by oxidation at the truncated chain of oxidized lipids, owing to their polarity, tend to reorient towards the aqueous phase. Orientation reversal leads to modification of layer structures, as demonstrated experimentally as well as through molecular dynamic (MD) simulations. Changes in oxidized molecule orientation lead to layer expansion and influence the layer’s properties (thickness, molecular ordering, rigidity, permeability) and stability (sometimes causing disintegration) by changing the molecular interactions within the layer (Sabatini et al. 2006; Wong-ekkabut et al. 2007; Jurkiewicz et al. 2012; Khabiri et al. 2012; Faye et al. 2013; Itri et al. 2014; Makky and Tanaka 2015).

Polar groups at the ends of truncated hydrocarbon chains modify the electrostatic properties of oxidized lipid species, thus leading to an increase or appearance of surface charges (Mosca et al. 2011).

The various roles played by sterols in biological membranes (Dahl and Dahl 1988; Yeagle 1988; Finegold 1993) justify the need for studying the effects of sterol presence on oxidation reactions. Cholesterol has been shown to strongly affect the properties of biological systems by causing an increase in layer rigidity and modifying the phase behaviour of lipid layers, at the molecular level by inducing ordering and condensation effects (van Blitterswijk et al. 1987; Veatch and Keller 2002; Silvius 2003; Róg et al. 2009; Bruning et al. 2010; Bennett and Tieleman 2013; Khelashvili and Harries 2013). Oxidation of cholesterol leads to various products, depending on the oxidant type and reaction conditions. Analytical (Ubhayasekera et al. 2004), thermodynamic (Lengyel et al. 2012) and biological (Schroepfer 2000; Hovenkamp et al. 2008; Vanmierlo et al. 2013) aspects of cholesterol oxidation have been discussed in the literature. In our studies, the most relevant outcomes were the results obtained for mixed systems, such as cholesterol/lipids under oxidizing conditions. Modification of phospholipid layer organization due to the presence of oxysterols has been studied by Theunissen et al. (1986) and recently by Kulig et al. (2015). The question of how lipid oxidation influences cholesterol behaviour in membranes has been investigated in papers by Jacob and Mason (2005) and Khandelia et al. (2014). The effect of cholesterol on lipid oxidation has been studied for mixed PC liposomes containing cholesterol or its ester after induction of oxidation by an AAPH radical initiator (Mosca et al. 2011). A protective effect of cholesterol during an ozone attack on the phosphatidyl glycerol layer has been studied using ionization mass spectrometry (Ko et al. 2012). All these studies have indicated protective effects of cholesterol against lipid oxidation, thus supporting a long-standing hypothesis about the antioxidant ability of this compound but also referring to the biological aspects of the problem (Smith 1991).

Many studies have characterized either lipid oxidation products or their mixtures with non-oxidized reactants. However, much less (experimental) work has been performed involving in situ tracking of the alterations in system properties during the reaction with oxidants (Borst et al. 2000; Megli and Russo 2008; Lilijeblad et al. 2010; Thompson et al. 2010; Qiao et al. 2013; Hemming et al. 2015).

Our two previous papers (Rudolphi-Skórska et al. 2014, 2016) have focused on the oxidation of layers of galactolipids, which are the main components of mitochondrial and chloroplast membranes (Gzyl-Malcher et al. 2008; Rudolphi-Skórska and Sieprawska 2016), by ozone. Comparison of the behaviours of monogalactosyldiacylglycerol (MGDG) and digalactosyldiacylglycerol (DGDG) clearly indicated a much higher resistance of the latter lipid to the destructive action of ozone dissolved in aqueous subphase. Unfortunately, because both these lipids have different unsaturation characteristics, it was difficult to unequivocally determine whether the shapes and sizes of polar groups or the distribution of double bonds in hydrocarbon chains should be used to explain differences. The more resistant DGDG, with two galactose groups forming polar regions, is composed of lipids, of which approximately 10% comprise asymmetrical lipids with one saturated palmitoyl residue. The presence of this fraction prompted us to investigate the effects of the structure of the hydrocarbon part on the oxidation of lipid layers. Thus, the aim of this work was to compare the effects of the oxidation of membrane-like structures (e.g. monolayers and liposomes) formed from lipids with the same choline polar group but with different structures and compositions of fatty acyl chains. Whether the presence of cholesterol influenced ozone-induced oxidation of cholines was also investigated.

## Materials and Methods

### Materials

Cholines: DOPC (18:1) 1,2-dioleoyl-*sn*-glycero-3-phosphocholine, POPC (16:0–18:1) 1-palmitoyl-2-oleoyl-*sn*-glycero-3-phosphocholine, soy PC, DPPC (16:0) 1,2-dipalmitoyl-*sn*-glycero-3-phosphocholine. Distribution of fatty acid chains in soy PC samples as given by the producer (Avanti Polar Lipids, Inc (USA/Canada)) is as follows: 16:0–14.9%; 18:0–3.7%; 18:1–11.4%; 18:2–63% and 18:3–5.7%.

Cholesterol was obtained from Sigma.

Phosphate salts used for buffer preparation was of chemical purity “POCh (Poland)”.

Solvents (chloroform, ethanol) of chemical purity were from “POCh (Poland)”.

Freshly deionized water was produced by HLP 5 apparatus “Hydrolab (Poland)”.

10 mM phosphate buffer pH 7 was used in all experiments.

### Methods of Ozonation and Determination of the Ozone Concentration

An Ozone generator FM 500 (prod. Grekos, Poland) supplied with oxygen and working on the principle of corona discharge was used as a source of ozone. The yield of ozone production was in the range of 200–500 mg/h. Buffer solution was saturated with ozone. Defined ozone concentrations were obtained by diluting the stock ozone solution.

Ozone concentration in buffer was determined by using the classic indigo method based on the decrease in the absorbance of indigo carmine (sodium indigodisulphonate) at *λ* = 287 nm (Bader and Hoigne 1981; Bader and Hoigne 1982; Majewski 2012). Discoloration of the dye was calibrated against the direct ozone UV peak at 250 nm, which was used as a primary standard with a molar extinction coefficient equal to 3200 M^−1^ cm^−1^(Sonntag and Gunten 2012). Due to loss of the ozone in bulk reactions leading to its decomposition, the ozone content in aqueous solutions decreases very rapidly. That's why samples for analysis were mixed with dye solution only at the moment of lipid deposition.

### Surface Pressure Isotherms

Surface pressure isotherms were measured using a Langmuir trough (Minitrough, KSV, Finland) with a total surface area of 243 cm^2^ with a Pt Wilhelmy plate used for surface tension detection. Measurements were made at a temperature of 25 °C at a constant rate of barrier movement equal to 5 mm/min, which, for most of the experiments, corresponded to the rate of area per molecule decrease in the range 2–5 Å^2^ molecule^−1^ min^−1^. An expanded layer of lipids was formed on a subphase containing ozone and a surface pressure was measured during the first 15 min at immobile barriers. Then, the movement of barriers was switched on to detect surface pressure isotherms, i.e. *π* = f(A) dependence, where *π* is the surface pressure and A is the area per molecule corresponding the two-dimensional layer density.

### Liposome Preparation and Characterization

Liposomes were prepared according to a classic, well-known procedure. The film of lipid, or lipid and cholesterol mixture, dissolved in chloroform, coating the wall of a round-bottomed glass tube was dried under an argon stream. The dried material was then hydrated with 0.01 M PBS buffer (pH 7), and vortexed. The resultant suspension was extruded using a polycarbonate membrane with a 200-nm pore size.

Liposome sizes and electrophoretic mobilities were determined using a Dynamic Light Scattering (DLS) method with a Malvern Zetasizer Nano ZS. The values of mobility were converted to zeta potentials with the Smoluchowski equation.

Liposome ozonation was accomplished through direct passage of ozone containing oxygen for 5 min. The final ozone concentration attained under these conditions was approximately 2 ppm.

### Surface Tension of Liposome Suspensions

The surface tension of liposome suspensions was determined by a classical stalagmometric method from the mass of 5 drops. The same liposome suspensions that were used for particle size and zeta potentials were used for surface tension measurements.

## Results

### Oxidation of One-Component Phosphatidylcholine Layers

**Fig. 1:**
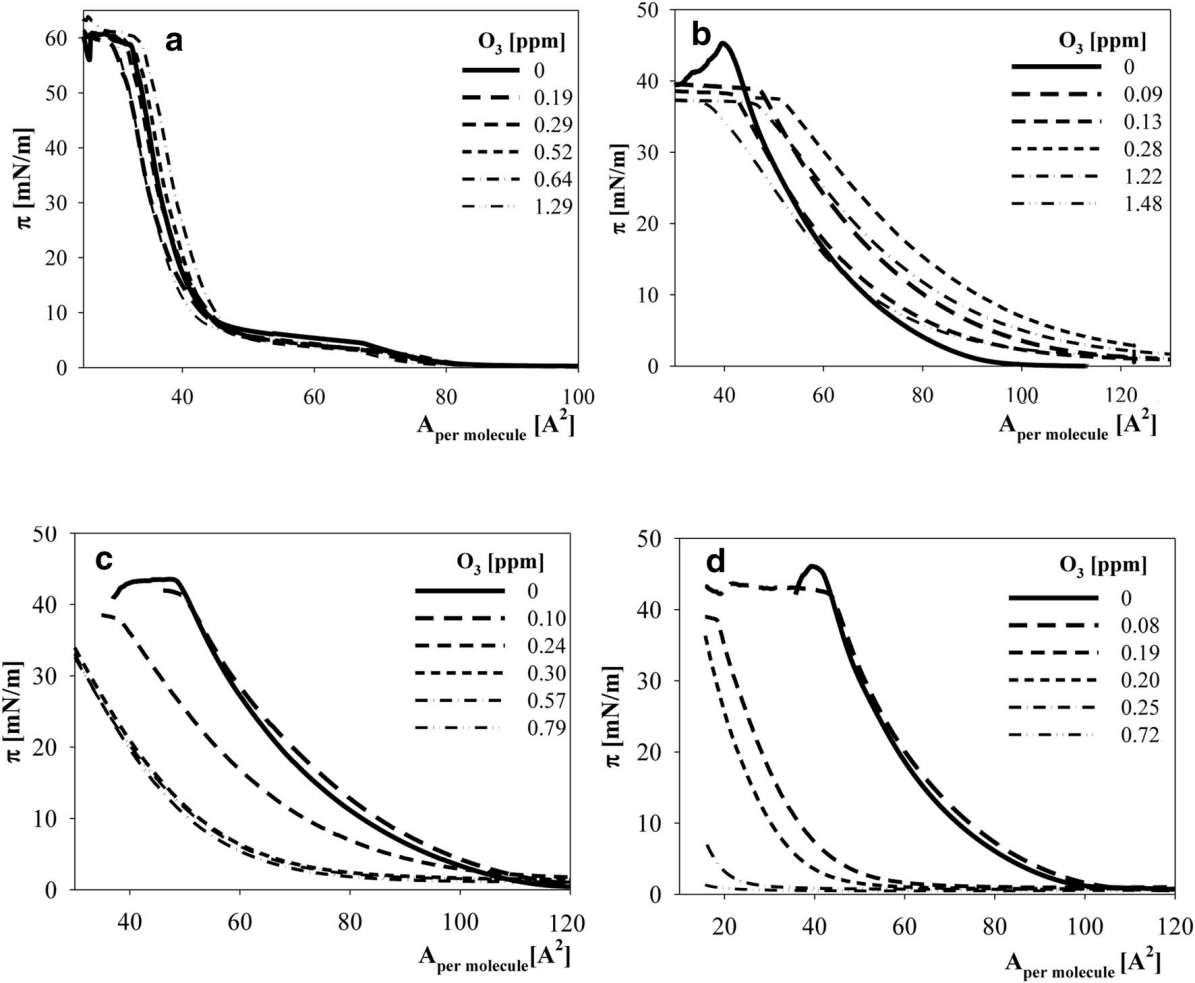
Surface pressure isotherms (*π* as a function of *A*_per molecule_) of layers of the studied cholines exposed to the action of ozone dissolved in a subphase (10 mM phosphate buffer pH 7) at various concentrations (indicated in a *graph legend*): **a** DPPC; **b** POPC; **c** soy PC and **d** DOPC

Surface pressure isotherms obtained for various ozone contents in the subphase are presented in Fig. 1a–d for studied cholines: DPPC (a), POPC (b), soy PC (c) and DOPC (d).

**Fig. 2:**
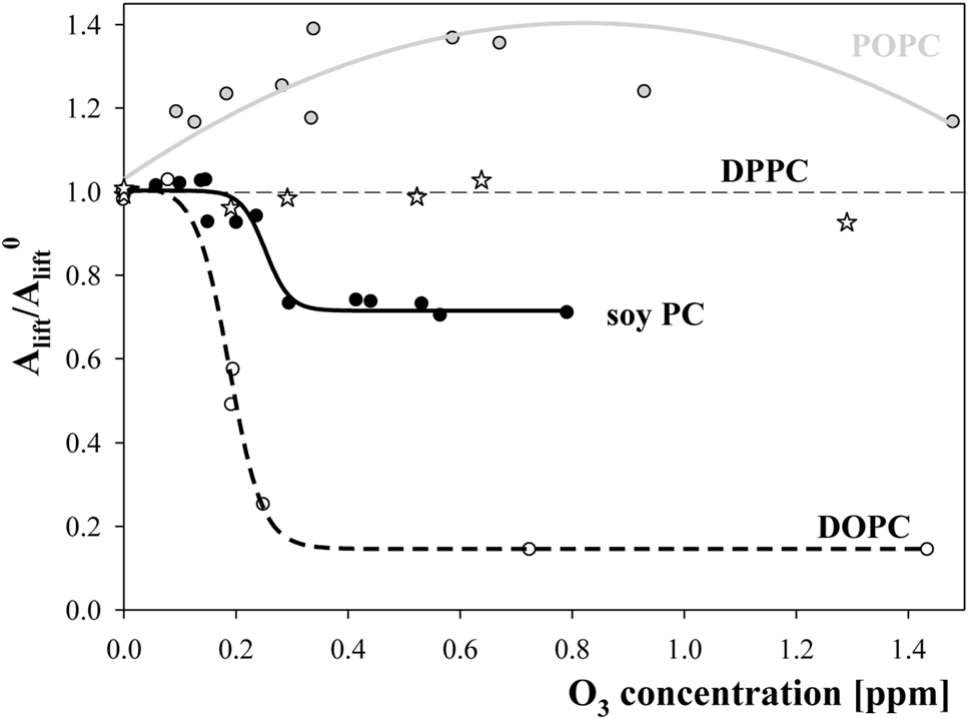
The dependence of the ratio of area per molecule corresponding 1 mN/m rise of surface pressure (area lift-off value) of oxidized layers relative to such a value characteristic for non-oxidized lipid (*A*_lift_/*A*_lift_^0^) on ozone concentration in a subphase for layers of DPPC—*open stars*, POPC—*filled grey circles*; soy extracted PC—*filled black circles*; DOPC—*open circles*. Lines do not present any physical model, were mathematically fitted and were drawn for the convenience of tracking parameter changes

The isotherms were analysed as described in Rudolphi-Skórska et al. (2014, 2016). The ability of layer formation was expressed as area per molecule for which the surface pressure increased by 1 mN/m (termed lift-off value) relative to a value characteristic of non-oxidized lipids (*A*_lift_/*A*_lift_^0^). The results obtained for all of the studied cholines are presented in Fig. 2.

As shown in Fig. 2, the behaviour of the studied lipids markedly differed. Surface pressure isotherms (Fig. 1a) and points (stars) in Fig. 2 for saturated DPPC clearly showed that the lipid did not change under the influence of ozone. Symmetric lipid (DOPC) and choline derived from soy gradually lost their ability to form layers as the concentration of ozone increased. At higher ozone concentrations, the ability of layer formation stabilized as indicated by constant plateau values of the ratio of lift-off areas being equal to 0.72 and 0.15 for soy-derived and symmetric DOPC, respectively. Ozonation of the POPC layer led to a shift in the surface pressure isotherms to the larger pressure values (Fig. 1b). To compare this effect with the effects obtained for soy PC and DOPC, the results were treated identically, and the ratio of *A*_lift_/*A*_lift_^0^ values rose with the ozone concentration in a non-monotonic manner with a maximal increase equal to 40% (the value obtained from fitted function).

**Fig. 3:**
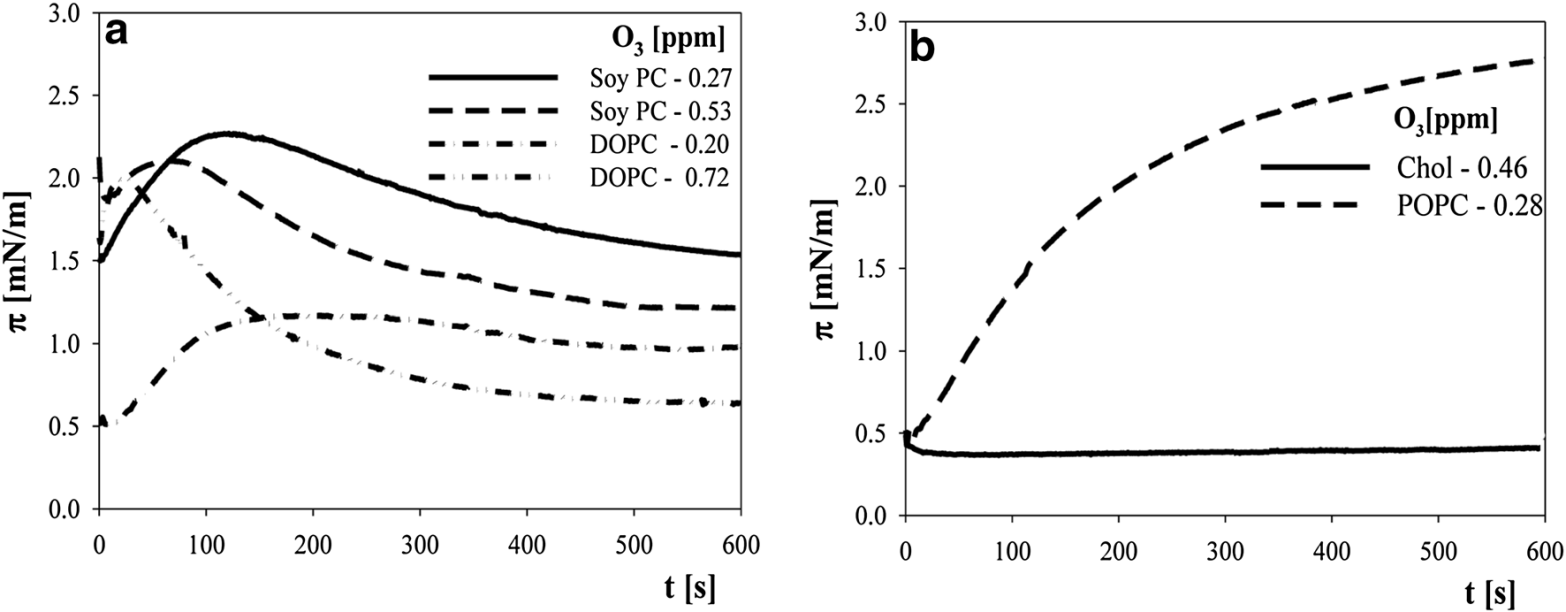
Time dependence of the surface pressure of diluted layers (just after lipid spreading) at a constant trough area (without layer compression). **a** Layers of soy PC (*solid line* 0.27 ppm O_3_; and *dash line* 0.53 ppm O_3_) and DOPC (*dash-dot line* 0.20 ppm O_3_ and *dash-dot-dot line* 0.72 ppm O_3_). **b** Layers of cholesterol at 0.46 ppm O_3_—*solid line* and of POPC at 0.28 ppm O_3_—*dash line*

Different behaviours of POPC were also found when the time dependence of the surface pressure of expanded layers (at a constant trough area) was examined (Fig. 3).

For synthetic (DOPC) cholines and cholines extracted from soy, the surface pressure of diluted layers after they were deposited onto a subphase changed in a non-monotonic manner, with the maximum value and its location dependent on ozone concentration. In case of POPC, the surface pressure increased monotonically with time, thus indicating interactions that expanded a layer. No measurable changes in the surface pressures of diluted cholesterol layers were observed.

### Oxidation of Mixed DOPC/DPPC Layers

**Fig. 4:**
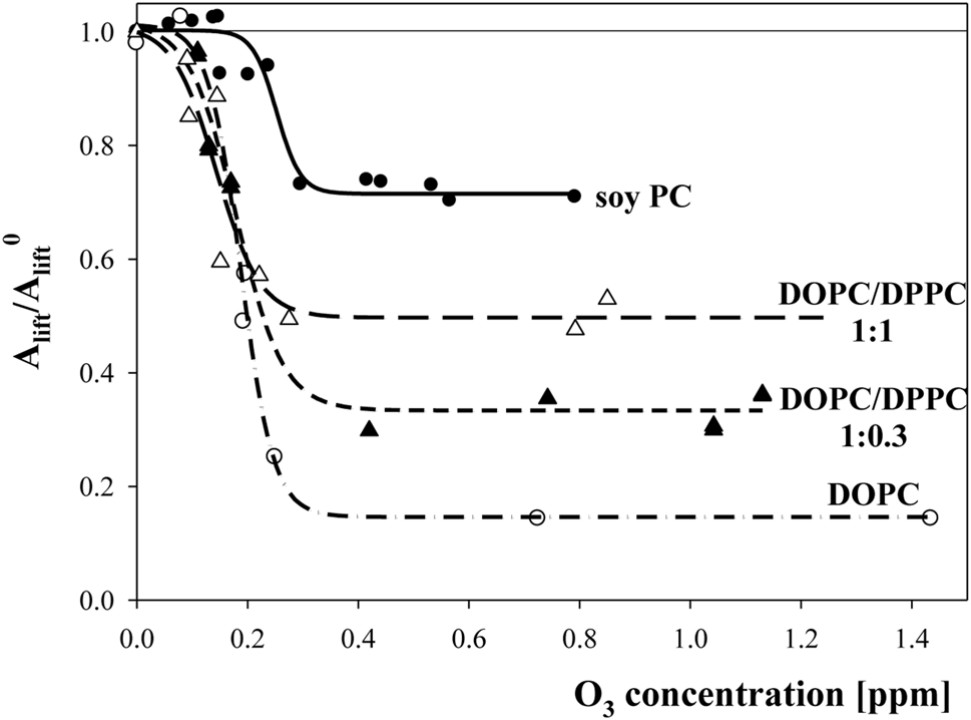
The dependence of lift-off areas ratio on ozone concentration in a subphase for layers of soy PC (*filled circles* and *solid line*), DOPC (*open circles* and *dash-dot line*) and DOPC/DPPC mixture of molar ratio 1:0.3 (giving the same unsaturation degree as soy PC)—*filled triangles* and *short dash line* and 1:1 DOPC/DPPC mixture—*open triangles* and *long dash line*. Lines were drawn as a guide for eyes

To determine what role unsaturation degree, which was defined as the fraction of fatty acid residues containing double bonds, plays in oxidation of lipid layers, measurements were performed for mixed layers of DOPC and DPPC at two molar ratios: 1:0.3 (to achieve an unsaturation degree similar to that of soy PC) and 1:1 (to observe an effect). As shown in Fig. 4, the presence of saturated lipid exerted a protective effect (as indicated by an increase in the plateau values of the ratio of lift-off areas) by stabilizing a layer containing unchanged molecules of saturated lipid. The jump of the ratio of lift-off areas (from 0.15 to 0.33) detected for the mixture of DOPC:DPPC of 1:0.3 molar ratio containing the same fraction of saturated fatty acyl chains as the soy-extracted lipid did not reach the value (0.72) observed for soy PC layers. However, the information provided by lipid producers about the fraction of unsaturated fatty acyl chains in soy PC did not specify whether this value referred to lipids in which double bonds were present in two or one fatty acid residue in the molecule.

**Fig. 5:**
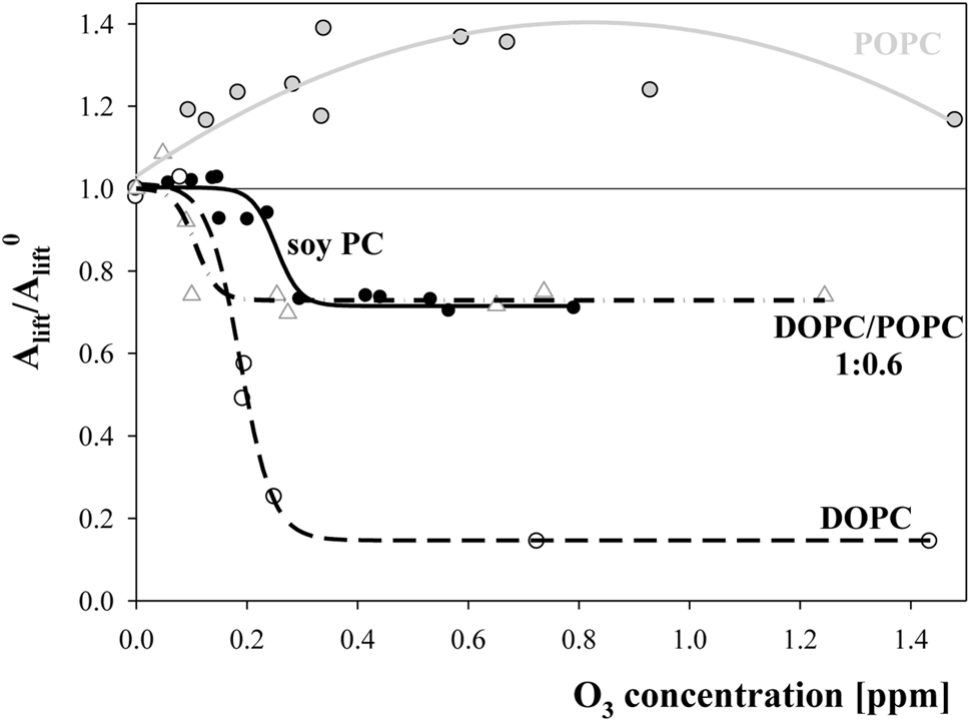
The dependence of the ratio of lift-off areas (*A*_lift_/*A*_lift_^0^) on ozone concentration in a subphase for layers of soy PC (*filled circles* and *solid line*), DOPC (*open circles* and *dash line*), POPC (*filled grey circles* and *solid grey line*) and DOPC/POPC mixture of molar ratio 1:0.6 (giving the same unsaturation degree as soy PC)—*open triangles* and *dash-dot line*. Lines were drawn as a guide for eyes

Thus, another attempt to generate a system that behaved similarly to soy PC was to mix POPC with an amount of bi-unsaturated DOPC, so that the resulting mixture had the same fraction of unsaturated fatty acyl chains. As shown in Fig. 5, this system behaved very much like choline extracted from soy. The only difference was that the mixed system started to react to the ozone presence at lower concentrations than did the one-component layer formed from soy PC.

### Ozonation of Cholesterol Layers

**Fig. 6:**
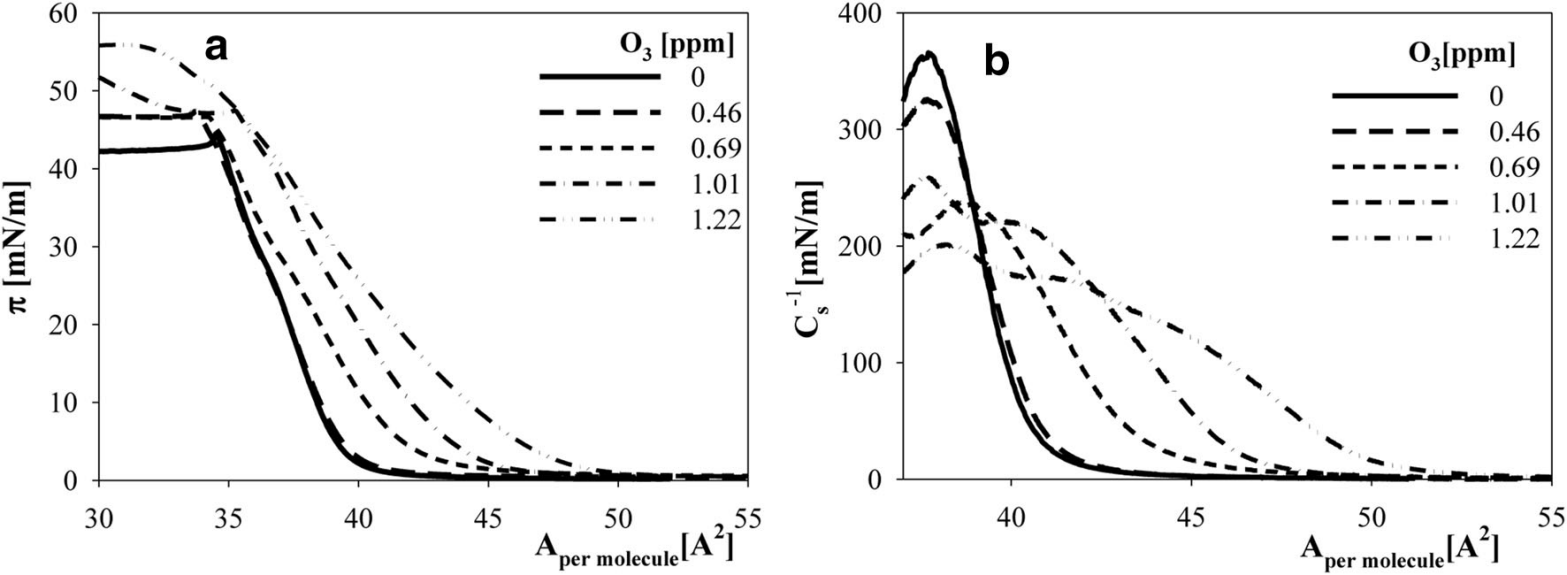
**a** Surface pressure isotherms (*π* vs area per molecule) of cholesterol spread on buffer containing various amounts of dissolved ozone: *solid line* 0, *long dash line* 0.46 ppm, *short dash line* 0.69 ppm, *dash-dot line* 1.01 ppm and *dash-dot-dot* 1.22 ppm. **b** Compressibility modulus $C_{\text{s}}^{ - 1}$ derived from these isotherms as a function of layer density expressed by the area per molecule

A similar series of measurements were done for lipids mixed with cholesterol. In the first step, effects related to one-component cholesterol layer oxidation were detected. In Fig. 6, surface pressure isotherms of cholesterol layers formed on buffer containing various amounts of ozone were presented.

Oxidation of cholesterol led to a noticeable increase in surface pressure over the entire range of layer densities (areas per molecule). However, this increase was imperceptible in the *π*(t) time dependence of the surface pressure of the diluted layer (not subjected to compression) at ozone content equal to 0.46 ppm (Fig. 3b). Surface pressure isotherms measured at rising ozone concentrations shifted in the direction of greater values of area per molecule that corresponded to the same *π* values (Fig. 6a). The effects of emerging oxidation products on layer properties were also presented according to the dependence of the compressibility coefficient, which was defined as $$C_{\text{s}}^{ - 1} = - {\raise0.7ex\hbox{${{\text{d}}\pi }$} \!\mathord{\left/ {\vphantom {{{\text{d}}\pi } {{\text{d}}\ln A}}}\right.\kern-0pt} \!\lower0.7ex\hbox{${{\text{d}}\ln A}$}}$$, on layer density (expressed by the value of A—area per molecule) (Fig. 6b). With increasing ozone levels, more diluted layers exhibited measurable resistance to compression, thus indicating growing repulsion in the layer.

**Fig. 7:**
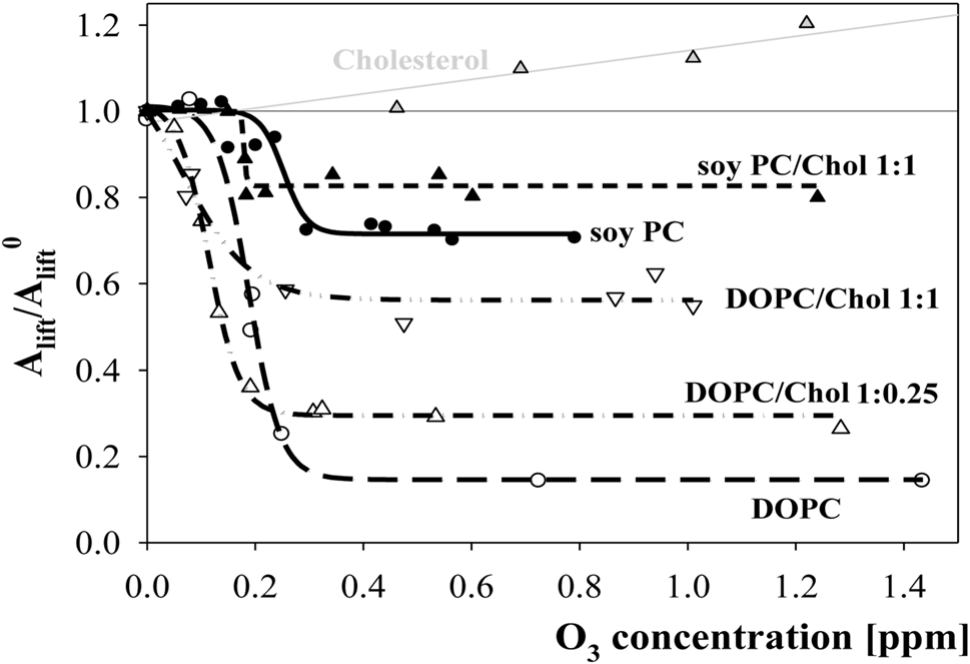
The dependence of the lift-off areas ratio (*A*_lift_/*A*_lift_^0^) on ozone concentration in a subphase for layers of soy PC (*filled circles* and *solid line*), DOPC (*open circles* and *long dash line*) and cholesterol *filled grey triangles up* and *grey line*. The effect of cholesterol addition represents data obtained for lipid/cholesterol mixtures: DOPC/Cholesterol of molar ratios 1:0.25—*open triangles up* and *dash-dot line*, and 1:1 ratio—*open triangles down* and *dash-dot-dot line*; and for soy PC/cholesterol of 1:1 molar ratio—*filled black triangles up* and *short dash line*

To standardize the presentation of the effects of ozone on lipid layers, including cholesterol, surface pressure isotherms were used to determine the ratios of lift-off areas per molecule for the defined ozone levels. The dependence of these values on ozone concentration for layers of cholesterol was almost linear (Fig. 7).

The effects of cholesterol presence on the oxidation of mixed PC/cholesterol layers, expressed by the dependence of the ratio of *A*_lift_ values on ozone concentration, are presented in Fig. 7. In the case of DOPC, for which two levels of cholesterol in the mixture were tested, its presence caused a significant decrease in material loss (which was manifested as an increase in the plateau value of the *A*_lift_ ratio). This effect was stronger when the cholesterol content in mixed layers was higher. A comparison of the effects of cholesterol (at 50% molar content) on the oxidation of bi-unsaturated DOPC and of natural origin soy PC indicated that cholesterol protects bi-unsaturated lipids more efficiently.

### Supporting Measurements of Liposome Zeta Potential (ζ) and Size

To better characterize the systems, sizes and electrophoretic mobility (recalculated to zeta potential) of liposomes formed from the studied lipids were determined with the DLS technique. The results are presented in Tables 1 and 2. Liposomes formed from the studied cholines exhibited negative zeta potentials. However, the small absolute values indicated a somewhat low surface charge for these objects. A slightly higher absolute value of the zeta potential was obtained for soya PC liposomes, possibly owing to oxidation of this lipid during its preparation from material of natural origin. After 5 min of ozone exposure to liposome suspensions (which assured an ozone concentration of approximately 2 ppm), the absolute values of the zeta potential increased, thus indicating the presence of charged groups on the surface of the liposomes.

**Table 1:** Electrostatics of the studied systems

Lipid	ζ [mV] of liposomes in pure buffer	ζ [mV] of liposomes after direct ozonation of suspension
Soy PC	−6.85 ± 1.12	−48.7 ± 4.05
DOPC	−2.29 ± 0.54	−38.4 ± 0.53
POPC	−2.47 ± 0.52	−37.95 ± 2.95
Cholesterol^a^		−76.5 ± 2.62

^a^Determined for particles obtained by dispersion of cholesterol film ozonized from a gas phase

**Table 2:** Relative changes of an average hydrodynamic diameter of liposomes *Z*_ave_ caused by suspension ozonation

Lipid	Relative change of *Z*_ave_ after ozone treatment (%)
DOPC	−62
Soy PC	+7
POPC	+45
DOPC + cholesterol 1:1	+1

Cholesterol does not form any liposomes and cannot be dispersed in aqueous media. However, after oxidation, it is possible, through intense vortexing of the hydrated film of oxidized cholesterol to enforce film dispersion enabling the measurements of the electrophoretic mobility of such particles. The results showed that particles of oxidized cholesterol were highly charged and exhibited zeta potentials with absolute values as high as 76 mV.

Although the liposomes prepared from cholines were quite uniform, with an average hydrodynamic diameter in the range of 150–210 nm (depending on preparation) and were characterized by a fairly low polydispersity index (PDI on average 0.15), after direct ozone application, they became polydisperse (PDI on average 0.6), thereby making analysis of the DLS signal ambiguous. Nevertheless, the average values of particle diameter *Z*_ave_, despite reservations regarding the signal analysis, clearly indicated the direction of the changes in particle size after oxidation.

The numbers presented in Table 2 are consistent with the results obtained for lipid monolayers. The most destructive effects of ozone, which were manifested by the greatest decrease in the ratio of *A*_lift_ values of DOPC layers, resulted in a dramatic decrease in the average diameter of liposomes prepared from this lipid and subjected to ozone treatment. In the case of POPC, the average diameter of liposomes increased. However, the possibility that particle aggregation might have led to the same effect cannot be excluded. The changes in the sizes of liposomes formed from the soy PC and 1:1 DOPC/Cholesterol mixture were insignificant.

A significant decrease in DOPC liposome average size indicated that after oxidation, a noticeable part of the mass leaves the liposome and dissolves in the aqueous solution. Because all oxidation products are organic compounds containing polar regions, it is probable that these products may be surface active. As such, their presence can be detected by measuring the surface tension of liposome suspensions. The presence of unmodified liposome particles did not influence the surface tension of pure buffer solution (within experimental error). Surface tensions of ozonized suspensions of all studied systems were noticeably lower and, when expressed by mass of drop (fixed at stalagmometer of tip radius equal to 0.14 cm), reached 60–90% (0.025–0.038 g) of the value for an unmodified suspension (0.042 g). However, the decrease in surface tension did not correlate with the effect of ozone on liposome size and the layer destruction indicated by *A*_lift_ values. In contrast to the sizes, which were essentially independent of the suspension concentration, the surface tension decrease should reflect the amount of lipids undergoing oxidation. Liposome preparation, including the extrusion (which may cause extensive loss of material deposited on polycarbonate membranes) does not allow for a quantitative association between surface tension values and lipid mass. Additionally, the surface activity of various oxidation products is not known. Thus, these results should be treated as a qualitative indication of the presence of low molecular size, surface active oxidation products dissolved in aqueous medium rather than as quantitative data characterizing a post-reaction system.

## Discussion

Numerous previous studies have indicated the importance of molecular structure in lipid oxidation (Lai et al. 1994; Lilijeblad et al. 2010; Reis and Spickett 2012; Faye et al. 2013; Qiao et al. 2013). Our two previous papers (Rudolphi-Skórska et al. 2014, 2016) and the results presented in this study indicate the usefulness of the lift-off area parameter for characterizing layers after oxidation and revealing factors that influence the process. The sigmoidal shape of the dependence of the *A*_lift_/*A*_lift_^0^ values (the ratio of *A*_lift_ the area per molecule, corresponding to a 1 mN/m increase in the surface pressure of a layer of oxidized lipids relative to that of a layer not subjected to oxidation) on ozone concentration seemed to be quite general, as observed for various unsaturated lipids. Two features of this relationship were relevant in the oxidation of lipid layers spread on an aqueous subphase, including the ozone threshold concentration at which this ratio suddenly changed and the constant, limiting (plateau) value of *A*_lift_/*A*_lift_^0^ reached at higher ozone concentrations. The first value reflects the resistance of a layer to oxidation, and the second value characterizes the oxidized system.

The most striking effect observed for the studied systems was the correlation between the plateau values of *A*_lift_/*A*_lift_^0^ reached in the range of high ozone concentrations and the composition of the hydrophobic parts of the lipid layers (Table 3). The highest limiting value of *A*_lift_/*A*_lift_^0^ (equal to 0.73) was found for layers formed from a DOPC/POPC mixture at a molar ratio of 1:0.6, which, despite the high degree of unsaturation (81.4%), had a substantial fraction of mono-unsaturated molecules containing saturated and unsaturated acyl chains in a single molecule (37.5%). A similar plateau *A*_lift_/*A*_lift_^0^ value (0.72) was obtained for soy PC (which is a mixture of various cholines for which only the total content of unsaturated fatty acid residues was provided by the producer), thus allowing us to assume that in that case, the fraction of mono-unsaturated lipid molecules should be similar as well. This assumption was supported by observations that the behaviour of the layer containing the same (81.4) percentage of unsaturated acyl chains but none of the asymmetric configurations (DOPC:DPPC of 1:0.3 molar ratio) was markedly different (*A*_lift_/*A*_lift_^0^ = 0.33), and oxidation led to noticeable destruction. Simultaneously, results from our previous paper (Rudolphi-Skórska et al. 2014) have indicated that layers of DGDG of an even higher unsaturation degree (95.2) but containing a certain amount of asymmetrically distributed unsaturated fatty acids (10% as specified by the producer), maintain their integrity better (lift-off areas ratio of 0.52).

**Table 3:** Parameters characterizing lipids’ unsaturation (unsaturation degree and fraction of mono-unsaturated molecules) and the effects of layers’ ozonation (ozone threshold concentrations and the plateau values of the ratio of lift-off areas per molecule, which corresponded to a 1 mN/m rise in the layer surface pressure for oxidized to non-oxidized lipids (*A*_lift_/*A*_lift_^0^))

Lipid	Unsaturation degree^a^ (%)	Fraction of asymmetric molecules^b^ (%)	O_3_ threshold concentration (ppm)	Plateau values of *A*_lift_/*A*_lift_^0^ ratio
MGDG^c^	100	0	0.21	0.16
DOPC	100	0	0.19	0.15
DGDG^c^	95.2	10	0.20	0.52
Soy PC	**81.4**	**unknown**	0.25	**0.72**
DOPC/DPPC 1:0.3 M/M	**81.4**	0	0.18	0.33
DOPC/DPPC 1:1	54.9	0	0.14	0.50
DOPC/POPC 1:0.6	**81.4**	**37.5**	0.11	**0.73**
Mixtures with cholesterol				
DOPC/Chol 1:0.25	100		0.12	0.30
DOPC/Chol 1:1	100		0.10	0.56
Soy PC/Chol 1:1	81.4		0.18	0.83

Bold refer to the systems considered in the discussion and are important to justify the thesis on the importance of distribution of double bonds in acid residues in the oxidative destruction of lipid layers

The values of ozone threshold concentrations and plateau values of the ratio of lift-off areas were obtained on the basis of functions mathematically fitted to experimental results

^a^Unsaturation degree calculated as a fraction of unsaturated acyl chains

^b^Asymmetric molecules, i.e. containing one saturated and one unsaturated fatty acid residue

^c^Results from authors’ previous paper (Rudolphi-Skórska et al. 2014)

MD simulations of DOPC membranes exposed to massive oxidation (Cwiklik and Jungwirth 2010; Khabiri et al. 2012) demonstrated bilayer disintegration that led to pore creation. The lowest limiting *A*_lift_/*A*_lift_^0^ value (0.15) was obtained for a DOPC monolayer that demonstrated similar disintegration. The results showed that the lipids with all the hydrocarbon chains containing double bonds behaved similarly despite their different polar groups (MGDG and DOPC).

Oxidative destruction of DOPC was weakened for mixtures containing fully saturated lipid (DOPC/DPPC), and this stabilizing effect increased along with the increasing fraction of saturated component in a manner similar to the results of Qiao et al. (2013). However, as was mentioned above, stability of oxidized layers formed from the mixture of unsaturated/saturated lipids (DOPC:DPPC of 1:0.3 molar ratio) was significantly lower (as expressed by the ratio of A lift-off values equal to 0.33) compared with the stability (*A*_lift_/*A*_lift_^0^ = 0.73) obtained for the mixed lipid layer with the same unsaturation degree (81.4) but containing lipids with mono-unsaturated molecules (DOPC:POPC of 1:0.6 molar ratio).

All of the present observations were further qualitatively supported by the results of liposome size and surface tension of oxidized liposome suspension measurements. A significant decrease in the average size of DOPC liposomes after ozonation suggested a meaningful loss of the material capable of bilayer formation. This effect was so large that it could not be connected to only bilayer reorganization. The soy lecithin liposome size reduction due to oxidation by 2,2′-Azobis(2-amidinopropane) dihydrochloride (AAPH) has also been described by Mosca et al. (2011). The opposite effect, an increase in POPC liposome size after ozonation, agreed with the observed increase in the area per molecule (at constant pressure) in monolayers subjected to oxidation, thus further supporting the descriptions in the literature of the behaviour of oxidized POPC molecules. Oxidized POPC molecules were anchored at the interface, owing to saturated fatty acyl chains with another truncated chain with a polar group at the end bent towards a polar phase (Sabatini et al. 2006; Wong-ekkabut et al. 2007; Jurkiewicz et al. 2012; Khabiri et al. 2012).

One should also take into account the noticeable decrease in the surface tension of the liposome suspension treated with ozone. While bearing in mind all reservations associated with the interpretation of surface tension data for oxidized liposome suspensions, the reduction of this parameter (registered for all studied systems) can be connected to the appearance of water-soluble, surface active species. From all of the observations made for monolayers and oxidized liposome suspensions, the conclusion about the dissolving low-molecular-weight oxidation products in the bulk solution seems to be justified.

As indicated by similar values in the zeta potentials of DOPC and POPC liposomes, charging induced by oxidation appeared to be quite similar for these cholines (as expected for lipids with the same polar groups), and these results were comparable to the data presented in Mosca et al. (2011) and Makky and Tanaka (2015). This trend indicates that the specific behaviour of PC layers may be connected to differences in the structure of the hydrophobic parts of the lipids.

The threshold values of ozone concentrations corresponding to the half-height of *A*_lift_/*A*_lift_^0^ = f(O_3_) dependence indicate the layers resistance to oxidation. For one-component lipid layers (including previously studied galactolipids), these threshold values were fairly similar and had an average of 0.22 ppm ± 12%. The exception was POPC, which reacted to the lowest applied ozone concentrations and exhibited the opposite behaviour with increased *A*_lift_/*A*_lift_^0^ values at higher ozone levels.

For two-component (DOPC/DPPC) layers, ozone threshold concentrations decreased as the content of the second ingredient increased. The shift was even larger when the second component had a different chemical nature (DOPC/Chol). These results correlated with the compressibility factor, which, for diluted mixed layers of unoxidized lipids, was generally smaller than that for layers comprising a single lipid type. Thus, the changes in molecular interactions in lipid mixtures (as indicated by *C*_s_^−1^ factor) may make the layer more sensitive to small ozone concentrations.

Interestingly, as shown in our previous paper (Rudolphi-Skórska et al. 2014, 2016), the presence of antioxidants that are soluble in an aqueous subphase (gallic acid) significantly influences the ozone threshold concentrations and does not affect the plateau values of *A*_lift_/*A*_lift_^0^. This result can be interpreted by attributing the role of this antioxidant to only a decrease in the amount of oxidizing radicals. The action of antioxidant present in the lipid phase (*α*-tocopherol), leads to an increase in the *A*_lift_/*A*_lift_^0^ plateau value and to a shift in the ozone threshold level, although the last change occurs to a lesser extent than the shift caused by water-soluble gallic acid.

Cholesterol protects lipid membranes through the inhibition of oxidant transport (Subczynski et al. 1989; Denicola et al. 1996) and through competitive oxidation engaging part of the oxidant (Ko et al. 2012). According to previous studies (Ubhayasekera et al. 2004; Yin et al. 2011; Lengyel et al. 2012; Kulig et al. 2015), non-enzymatic oxidation of cholesterol leads to the formation ring-oxidized sterols, which, by altering the molecular interactions within membranes, modify membrane physical properties (e.g. transport, permeability, stiffness) and thus their functionality.

Under oxidizing conditions, cholesterol behaves differently from phospholipids. Molecules of oxidized cholesterol remain in a layer, thereby causing its expansion (Fig. 6). As indicated by the high absolute values of zeta potentials of oxidized cholesterol particles (Table 1), oxidation products are charged, and, owing to electrostatic repulsion, may be at least partially responsible for the increased values of area per molecule at a constant surface pressure or increased surface pressure in layers of constant density.

As mentioned above, the presence of the second component in choline/cholesterol mixtures resulted in a decrease in the ozone threshold level, thus indicating a higher sensitivity of the mixed layer to smallest tested amount of ozone.

The increased plateau *A*_lift_/*A*_lift_^0^ values obtained for choline/cholesterol mixtures indicated a stabilizing effect exerted by cholesterol in ozone-treated mixed layers. As one would expect, the greatest influence of cholesterol occurred for the layer of lipid that was the least stable against oxidation, i.e. DOPC. Limiting values of the ratio of lift-off areas, characterizing the post-reaction state of the DOPC/cholesterol layers, increased by 2.0- and 3.8-fold (compared to DOPC) depending on the cholesterol content. Additionally, there was a 15% increase in the limiting *A*_lift_/*A*_lift_^0^ value that was observed for a 1:1 mixture of relatively stable soy PC with cholesterol.

The lack of measurable changes in the size of the DOPC/cholesterol liposomes exposed to direct ozone treatment indicates also the stabilizing action of cholesterol (Table 2). These observations were in agreement with the measurements made by Mosca and colleagues (Mosca et al. 2011).

In interpreting the observed effects, the fact that they represent various changes occurring in the course of a reaction with ozone should be considered. The organization of lipid membranes is greatly influenced by cholesterol, which significantly modifies the phase equilibria in mixed bilayers, depending on the cholesterol content (Khelashvili and Harries 2013). Jacob and Mason (2005), through small-angle X-ray diffraction measurements, have found that cholesterol, when randomly distributed under weak oxidizing conditions, undergoes self-association in the presence of highly peroxidized lipids, thus forming membrane-restricted domains that produce an overall decrease in bilayer width. Interpretation referring to layer organization has also been applied to the results of MD simulations, supported by DLS liposome size measurements (Khandelia et al. 2014) conducted for bilayers composed of unmodified and oxidized lipids and cholesterol. The authors have claimed that cholesterol protects phospholipid bilayers from the influence of lipid oxidation products by sequestering conical-shaped oxidized lipid species away from phospholipids, owing to shape complementarity, which induces the self-assembling of cholesterol and oxidized lipids into bilayers.

The opposite situation, i.e. the effects of oxysterols on lipid (POPC) layer organization and properties, has been discussed in (Kulig et al. 2015). The results obtained through numerous techniques and simulations show that ring-oxidized sterols disturb the membrane structure by increasing the mobility of lipid carbonyls and decreasing membrane order.

In summary, the presented results do not specify the various stages of the ozone attack on lipid layers. However, by exposing the system in its entirety to oxidation, the changes in the properties of lipid layers included all the effects resulting from the oxidation reaction occurring in situ (i.e. its kinetics, system composition changes occurring along with reaction progress and affecting molecular interactions within a layer). Overall, our observations focused on all the consequences of oxidation in terms of layer stability and properties. In our opinion, the most important message is the existence of a crucial relationship between the distribution of double bonds in the hydrophobic parts of lipids and the membrane resistance to oxidation.

Although the present results were obtained for simplified model systems, the information may be valuable for native membranes that must maintain their continuity and functionality under severe oxidative stress, which is key for keeping cells alive. In the course of the reaction, the emergence of oxidized products that modify membrane structures influences the interactions of lipid components with membrane proteins, whose operation (i.e. transport of various substances to/from the cell, receipt and propagation of signals and enzymatic activity) may also be affected.

Oxidation-induced modifications of membrane properties not only influence processes directly related to and localized within the membrane but also affect many physiological processes in cells that are important for the regulation of gene expression. Therefore, all changes occurring within the membrane are important for the metabolism of whole organisms.

## Conclusions

1. The degree of ozone-induced degradation of lipid layers is largely dependent on the composition and structure of the hydrophobic parts of lipids. The unsaturation degree (the fraction of unsaturated fatty acid residues usually used as an indicator of the lipid vulnerability to oxidation) as well as the distribution of double bonds between fatty acyl chains of lipid molecules (i.e. the content of asymmetrical unsaturated lipid molecules containing one saturated hydrocarbon chain) are relevant.
2. The above conclusion was reached on the basis of the behaviour of layers of phospholipids with the same polar group (choline) and different fatty acid residues, including bi-unsaturated DOPC, mono-unsaturated POPC and phosphatidylcholine of natural (soy) origin. The most destructive effects of reactions with ozone were found for DOPC with both unsaturated fatty acyl chains. The effects were smaller for soy PC of an unknown fraction of asymmetrical lipid molecules, which, however, were characterized by a lower degree of unsaturation compared with that of DOPC. Ozonized layers of mono-unsaturated POPC did not exhibit any disruption, even though their modified properties indicated structural changes caused by the appearance of oxidation products.
3. The behaviour of systems composed of the studied lipids mixed with saturated DPPC (which alone did not react with ozone) demonstrated a protective effect stabilizing layers after oxidation. By mixing DOPC and DPPC, we were able to create systems with defined degrees of unsaturation; however, these systems did not contain any asymmetrical molecules with one saturated hydrocarbon chain. The obtained results indicated the role of unsaturation degree in the stabilization of ozonized layers.
4. The oxidation of DOPC and POPC mixtures of composition characterized by the same unsaturation degree as soy PC, caused layer destruction to an extent that was almost identical to that observed for soy PC. This similarity suggested that PC with a natural origin should contain comparable amounts of asymmetrical molecules to those in the mixture consisting of DOPC and POPC at molar ratios of 1:0.6.
5. Previous results obtained for MGDG and DGDG (Rudolphi-Skórska et al. 2014) fit well with the results presented here for phosphatidylcholines and additionally support that the presence of asymmetrical lipids has a decisive role in oxidized layer stability with much smaller, if any, influence of the polar parts of lipid molecules.
6. The stabilizing effects of cholesterol expressed by an increase in plateau values of *A*_lift_ ratio were also determined. For 1:1 molar mixtures of studied cholines with cholesterol, the plateau *A*_lift_ ratios were equal to 1.16 (0.83 for mixture vs 0.715 for pure soy PC) and 3.40 (0.50 for mixture vs 0.15 for pure DOPC).
7. The results obtained for lipid layers were supported by measurements of liposome sizes, zeta potential and the surface tension of suspensions, thus qualitatively confirming the conclusions derived from experiments with lipid monolayers.
